# Anticipation of thermal pain in diverticular disease

**DOI:** 10.1111/nmo.12790

**Published:** 2016-03-11

**Authors:** J. K. Smith, L. Marciani, D. J. Humes, S. T. Francis, P. Gowland, R. C. Spiller

**Affiliations:** ^1^ Nottingham Digestive Diseases CentreSchool of MedicineUniversity of NottinghamNottinghamUK; ^2^ Sir Peter Mansfield Magnetic Resonance CentreSchool of Physics and AstronomyUniversity of NottinghamNottinghamUK; ^3^ Nottingham Digestive Diseases Biomedical Research UnitNottingham University HospitalsUniversity of NottinghamNottinghamUK

**Keywords:** anticipation, diverticular disease, functional MRI, pain

## Abstract

**Background:**

The relative importance of peripheral nerve injury or central pain processing in painful diverticular disease (DD) is unclear. Functional magnetic resonance imaging (fMRI) has demonstrated that dysfunctional central pain processing predominates in irritable bowel syndrome (IBS). This study aims to identify anticipatory changes in symptomatic DD (SDD) compared to asymptomatic DD (ADD) and IBS patients.

**Methods:**

Gastrointestinal symptoms and somatization were evaluated via the Patient Health Question‐12 Somatic Symptom and the SDD group divided into low (≤6 [LSDD]) and high (≥7 [HSDD]) somatization. Cued painful cutaneous thermal stimuli were delivered to the left hand and foot during fMRI. Fixed effect group analysis of the ‘cued’ anticipatory phase was performed.

**Key Results:**

Within the right posterior insula, greater deactivation was found in the ADD compared to other groups. In emotion processing centers, anterior and middle insula, greater activation was identified in all patient compared to the ADD group, and in LSDD compared to IBS and HSDD groups. In comparison, amygdala deactivation was greater in ADD than the IBS and HSDD groups, and in LSDD *vs* HSDD groups. Descending nociceptive control centers, such as the superior medial frontal and orbitofrontal cortex, also showed greater deactivation in the ADD and LSDD compared to the HSDD and IBS groups.

**Conclusions & Inferences:**

The HSDD group have altered anticipatory responses to thermal pain, similar to IBS group. The LSDD are similar to ADD group. This suggests underlying differences in pain pathophysiology, and the need for individualized treatment strategies to target the cause of their chronic pain.

**Abbreviations**
ACC: anterior cingulate cortex
ADD: asymptomatic diverticular disease
aINS: anterior insula
AMYG: amygdala
CHEPS: Contact Heat‐ Evoked Potential Stimulator
DNIC: descending noxious inhibitory control
EPI: echo‐planar imaging
fMRI: functional magnetic resonance imaging
GLM: general linear model
HAD: Hospital Anxiety and Depression Score
HSDD: high somatization score symptomatic diverticular disease
IBS: irritable bowel syndrome
INS: insula
lat: lateral
LSDD: low somatization score symptomatic diverticular disease
MCC: mid‐cingulate cortex
mPFC: medial prefrontal cortex
PCS: pain catastrophizing score
PFC: prefrontal cortices
PHQ‐12 SS: Patient Health Questionnaire‐12 Somatic Symptom
PHQ‐15: Patient Health Questionnaire‐15
pINS: posterior insula
RFX: random effects
SDD: symptomatic diverticular disease
SPMMRC: Sir Peter Mansfield Magnetic Resonance Centre
THAL: thalamus
VAS: visual analog score

**Key Points**

- Symptomatic diverticular disease patients can be separated into low (LSDD) and high (HSDD) somatization groups based on Patient Health Questionnaire‐12 (PHQ‐12 SS)
- During anticipation of pain greater deactivations occur in somatosensory, emotional, and descending noxious inhibitory control pain regions in the asymptomatic (ADD) compared to the symptomatic diverticular disease (SDD) and irritable bowel syndrome (IBS) groups
- There are fewer anticipatory differences between the ADD and LSDD and the IBS and HSDD groups, suggesting that the LSDD and HSDD grouping identifies DD patients with predominantly peripheral *vs* central factors, respectively.

## Introduction

Colonic diverticulosis (DD)^1^ is the most common structural abnormality of the colon, yet our understanding of how it causes symptoms is rudimentary. It is responsible for substantial morbidity and mortality, with 254 179 hospital admissions, 1 493 865 outpatient visits in 2002 in the USA,^2^ and 23 000 deaths per year in Europe.^3^ Studies suggest its incidence and/or complications^4^ are increasing^5^, ^6^, ^7^, ^8^ with an associated increase in cost.^9^

While the acute complications of DD are well recognized, chronic painful symptomatic diverticular disease (SDD), in the absence of acute diverticulitis, is a poorly understood complication which causes much distress. Risk factors for developing SDD include a previous episode of inflammation, such as diverticulitis, adverse psychological conditions,^4^ low levels of physical activity,^10^ high BMI,^11^ and smoking.^12^ We and others have previously shown that SDD is associated with changes in colonic innervation, including increases in tachykinins, substance P, acetylcholine, nitric oxide, endocannabinoids, and galanin in the submucosal plexus and circular muscle,^13^, ^14^ and increases in neuronal angulation and density.^13^ This suggests an underlying peripheral nerve response to inflammation, which by analogy with animal studies,^15^ would be expected to result in hypersensitivity to colorectal distension.

Like SDD, there is a well‐recognized subgroup of IBS, postinfectious IBS (PI‐IBS), where symptoms and mucosal changes occur after an inflammatory episode, such as gastroenteritis.^16^ However, psychological factors, such as neuroticism and depression increase the risk of developing PI‐IBS.^17^ Anxiety, depression, and somatization, are also important to a lesser extent in SDD, and other conditions. The Patient Health Questionnaire‐12 Somatic Symptom scale (PHQ‐12 SS) has been used to assess somatization in SDD and IBS.^18^ The PHQ‐12 SS is a modified version of the PHQ‐15, but with three questions concerning gastrointestinal symptoms excluded. Using a PHQ‐12 SS score of greater than six, 67% of IBS and 55% of SDD patients have values above the normal range.^18^

These findings suggest that in both groups there are some individuals who have a predominantly postinflammatory disorder, possibly mediated by peripheral nerve hypersensitivity, with few other symptoms (i.e., low somatization or PHQ‐12 SS score), and others who have a more central cerebral‐based pain processing disturbance as indicated by a high somatization or PHQ‐12 SS score.^19^

There have been no studies characterizing central brain responses in DD, but we have previously found that somatization is a risk factor for developing DD symptoms, suggesting that alterations in pain processing may be present.^4^ Alteration in somatization and pain processing has been identified in patients with IBS.^20^, ^21^ Although there are some similarities between DD and IBS, there are also key differences, such as older age of onset, lesser female predominance, and the lack of pain relief after defecation in SDD.^4^, ^22^ We hypothesize that the SDD group, like IBS, can be separated into low (low somatization score SDD; LSDD) and high (high somatization score SDD; HSDD) somatizers based on the PHQ‐12 SS score.

Prior studies have suggested that the anticipation of pain may involve a network of brain areas. This includes the posterior insula, and anterior cingulate cortex (ACC) and anterior insula, key areas associated with somatosensory and emotional pain processing pathway and interoception.^23^, ^24^ In addition, affective brain regions of orbitofrontal cortex and amygdala, and the mid‐cingulate cortex (MCC) are of interest as these are implicated in fear processing and nociception.^25^ So also is the ventrolateral prefrontal cortex which is involved in the cognitive modulation of pain^26^ and diffuse noxious inhibitory control (DNIC)^27^ in bottom‐up modulation of the neural activity underlying pain together with the anterior and MCC.

The aim of this study was to identify differences in cerebral responses to the anticipation of pain in the SDD group based on PHQ‐12 SS somatization score, and to determine if high somatization was associated with an IBS‐like response to anticipation of pain.

## Materials and Methods

### Subjects

Study participants with IBS, ADD, and SDD were identified and recruited from gastrointestinal medicine and surgery clinics and databases of interested patients held at the Nottingham Digestive Diseases Centre (NDDC) NIHR BRU. Confirmation of the participants’ gastrointestinal diagnosis and the initial screening questions for inclusion and exclusion criteria (Table 1) were addressed by structured telephone questionnaire, before the study day. Detailed inclusion and exclusion criteria have been included in Table 1 to provide clarity in comparison with other studies as suggested in a recent review.^35^ Since our SDD patients had a range of bowel habits our IBS cohort was recruited solely on the basis of the Rome III criteria of recurrent abdominal pain/discomfort regardless of bowel habit. All study participants had structural imaging as part of their hospital diagnosis, either with flexible sigmoidoscopy or colonoscopy, CT, or barium enema. The study was approved by the Nottingham Regional Ethics Committee (09/H0403/43).

**Table 1:** Study Inclusion and Exclusion criteria

Inclusion criteria	
Participants must have either	Symptomatic diverticular disease with short‐lived recurrent abdominal pain on 3 or more days a month and at least one or more colonic diverticulum identified on endoscopy, barium enema, or CT scan
	Asymptomatic diverticular disease, with no abdominal pain and at least one or more colonic diverticulum identified on endoscopy, barium enema or CT scan
	Irritable bowel syndrome, which has been diagnosed by a gastroenterologist at the hospital using ROME II or III criteria
Age	18–85 years
Handedness	Right
Informed consent	Yes
Exclusion criteria	
General:	Pregnant or lactating women
	Severe co‐morbidity; for example, heart failure, respiratory failure, alcoholism, or drug dependence
	Participation in any other study on Nottingham University campus in the last 3 months
	No restrictions on the use of HRT, contraceptives medications, or timing of menstrual cycle with the study day were imposed
Metallic implants or objects	Cardiac pacemaker
	Implanted cardiac defibrillator
	Metallic heart valves
	Aneurysm clips
	Carotid artery vascular clamp
	Neurostimulator
	Insulin or infusion pump or implanted drug infusion device
	Non‐removable cochlear, otologic, or ear implant
	Shot or shrapnel inside the body
	Metallic fragments in the eye
Medications	Inability to stop NSAIDs (non‐steroidal anti‐inflammatory agents), antibiotics or immunosuppressant drugs or taking antiepileptic, gabapentin, long‐term opiates, or antipsychotic medications 28
	Participants taking ondansetron were included in the study, but the medication was not taken until after the study 29, 30, 31, 32
	No exclusions for patients taking antihypertensive medications, diuretics, alcohol,^33^ or caffeine 34 prior to the study
Inflammatory conditions	Presence of other gastrointestinal conditions such as ulcerative colitis, Crohn's disease and Celiac disease, malignancy, cirrhosis, current hematological malignancy, untreated peptic ulcer disease, Polymyalgia rheumatic
Abdominal surgery	Previous abdominal surgery (other than appendectomy, hysterectomy, cholecystectomy and sterilization, hernia repair)
Neurological conditions	Previous diagnosis of neurological conditions, for example, stroke, cerebral malignancy, essential tremor, Parkinson's disease and Parkinson plus syndromes, motor neuron disease, dementia, storage disorders, Wilsons disease e.t.c. Peripheral neuropathy (e.g., diabetic, alcohol, stroke)
Other	Claustrophobia, broken skin

### Sample size estimation

Based on the literature and our previous work,^36^, ^37^ we estimated that to show a >30% difference in functional magnetic resonance imaging (fMRI) response between groups, which is conventionally considered to be the minimal clinically significant difference, with a 80% power using alpha <0.05 would require *n* = 12 subjects. We aimed to recruit 20 subjects in each group to allow for a possible 40% drop‐out rate and/or poor compliance with fMRI protocol.

### Questionnaires

Participants completed validated questionnaires on gastrointestinal habits,^14^ Hospital Anxiety and Depression scores (HAD),^38^ somatic symptoms (PHQ‐12 SS)^18^, ^39^ and pain catastrophizing score (PCS)^40^ the day before the scan session. None of the participants’ usual medications or food were withheld before the visit except for ondansetron (IBS participants).^30^, ^41^

### Study protocol

**Figure 1:**
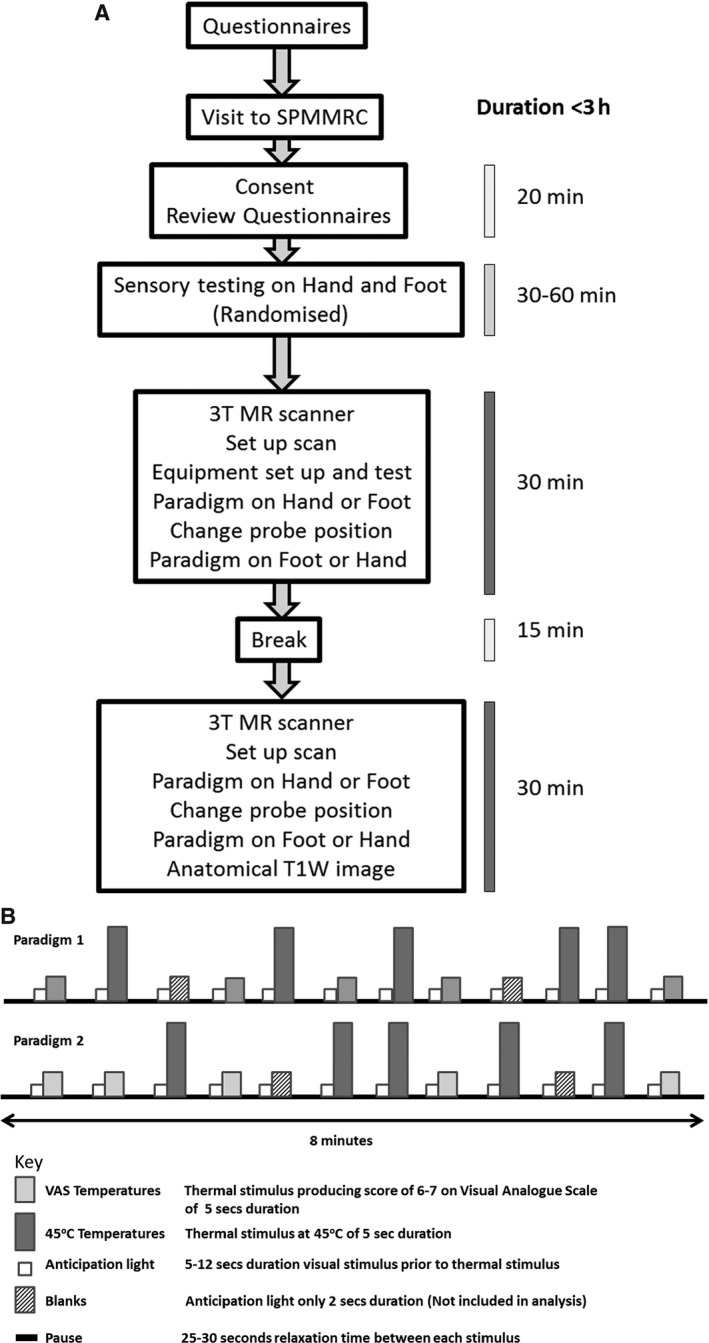
(A) Study flow diagram and Basic. (B) Paradigm design.

A Medoc PATHWAY System (Medoc, Israel) was used to deliver thermal stimulation using a MR‐compatible CHEPS (Contact Heat‐Evoked Potential Stimulator) 27‐mm‐diameter thermode probe (Fig. 1A and B). Although no formal handedness questionnaires were performed, participants were asked to identify their dominant hand based on the types of activities they preferentially used it. The thermode was placed on the dorsum of the left non‐dominant hand or foot and maintained in place using a Velcro strap and tubi‐grip bandage. Thermal sensitivity measures were undertaken outside of the MR scanner. To identify the unpleasant but tolerable temperature at which to perform the study (moderate pain temperature [MPT]), participants were asked to rate a series of temperature on visual analog score (VAS). This is a score of 0 to 10, where 0 = ‘no pain’ and 10 = ‘most severe pain ever experienced’. This test was repeated with different temperature until a VAS score of 6–7 was given. This temperature was designated to be the MPT and used as the individualized painful stimulus for the study.

Functional magnetic resonance imaging data were acquired on a 3 T Philips Achieva MR scanner using a 32‐channel receive coil. Participants viewed a projection screen in front of the magnet bore using a mirror attached to the receive coil. Participants were instructed to focus on the small blue cross projected on the screen which changed to a white cross to give a visual anticipation ‘cue’ prior to any stimulus. Participants were asked to pay attention to the screen and to the heat stimulus when delivered. Two pseudo‐randomized thermal stimulation paradigms shown in Fig. 1B were delivered based on published studies.^25^, ^26^, ^27^, ^28^ Each paradigm was of 8–9‐min duration and applied to the left hand and foot. The paradigms included the MPT and a standardized temperature of 45 °C for each participant. However, not all participants could tolerate this temperature, and it was reduced by 0.5–2 °C for some participants. Stimuli which had an anticipation cue of only 2–3 s were also incorporated into the paradigms, to prevent participants predicting the commencement of each stimulus. These shortened cues and stimuli were called ‘blanks’. The order of which paradigm was applied to which body site was randomized prior to commencement of the study. A 15‐min break half way through the study was used to prevent fatigue and reduced concentration. The participants then returned to the scanner, where the study was completed.

### fMRI data acquisition

The image acquisition used for the fMRI study was a single‐shot, double‐echo, gradient echo echo‐planar imaging (EPI), with echo times (TE) of 25/50 ms, a 80 × 77 matrix of 40 contiguous 3‐mm isotropic slices covering the whole brain. One hundred and seventy‐seven dynamic scans were acquired during a single thermal stimulation paradigm in which thermal stimulation was applied to the foot or the hand. Axial images were aligned along the AC–PC axis to aid the minimization of susceptibility artifacts in the orbito‐frontal cortex arising from the nasal cavity. Other scan parameters were SPIR fat suppression and a 80° flip angle to match the Ernst angle for the repetition time (TR) of 3s. A T_1_‐weighted MPRAGE anatomical image (256 × 256 matrix, 160 slices, 1‐mm isotropic resolution, TE/TR = 3.8/8.2 ms, 8° flip angle, 5 min acquisition time) was collected at the end of the fMRI session.

### Psychometric data analysis

A PHQ‐12 SS score of ≤6 (Low somatization score SDD; LSDD) or ≥7 (High somatization score SDD; HSDD) was used to separate the SDD patients into two groups based on our previous work.^18^ Participant questionnaire data were analyzed in SPSS (version 15; IBM, Portsmouth UK) and GraphPad Prism (Version 5; San Diego, CA, USA). A Shapiro–Wilks test was performed on the questionnaire data to test for normality. Age, bowel frequency and HAD scores of depression and anxiety were non‐parametric, the remaining numerical data were normally distributed. Group data were compared using a Fisher‐exact test, paired *t*‐test or Mann–Whitney *U* depending on the result of the Shapiro–Wilks test, with differences being considered significant at a *p*‐value of <0.05.

### fMRI data analysis

All fMRI images were analyzed using SPM8 (http://www.fil.ion.ucl.ac.uk/spm). Images were corrected for movement, slice timing, and normalized to the MNI template, following by spatial smoothing (8‐mm kernel). A general linear model (GLM) was used to model the heat stimuli and cue period, with each being convolved with canonical hemodynamic response function. For each participant, motion parameters during each paradigm were used as covariates of no interest. Blank stimuli were not modeled within the analysis (See Fig. 1B). First‐level fixed effects analysis was performed for each participant. Anticipation data from ‘blank’ stimuli were not analyzed. Data included in the second‐level analysis was based on completed questionnaire data being obtained, satisfactory data collection for all of the paradigms and anatomic sites. This resulted in 14 participants per analysis group. Second‐level random effects (RFX) group analysis for the anticipation cue stimulus (uncorrected *p* < 0.001, voxel threshold 5) was performed. In addition, a two‐sample *t*‐test was performed to compare the response to ‘cue’ events between each group (IBS, ADD and SDD) at an uncorrected *p* < 0.05, and cluster threshold of 5. All active clusters were identified using the WFU Pick Atlas (version 2.4).^42^ Covariates of interest such as anxiety and depression scores on the HAD questionnaire, total PCS, and PHQ‐12 SS were also included in the GLM and significant brain activity which correlated with these measures was assessed.

## Results

### Demographics

**Figure 2:**
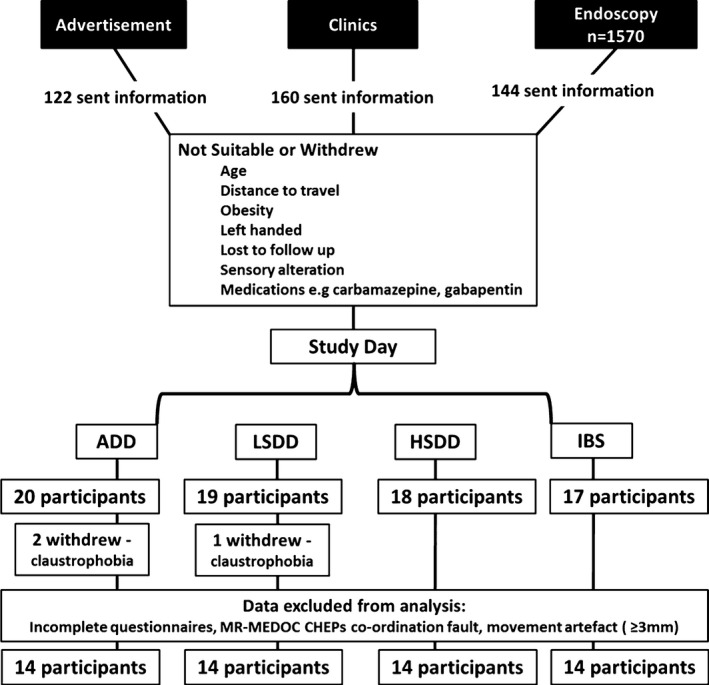
Diagram to illustrate participant recruitment. Three participants withdrew from the study: two after the sensory testing and before scanning and one at the break after the first scanning session.

Four hundred and twenty‐six potential participants were sent standardized information, with 74 participants being recruited; 18 with IBS, 20 with ADD, and 36 with SDD, 3 participants withdrew from the study (two from the ADD and one from the LSDD group) (Fig. 2). Key demographic details for the second‐level RFX analysis subset, are shown in Table 2. Significant differences in age were found between these groups with the LSDD and ADD groups compared to the HSDD and IBS groups, who were younger.

**Table 2:** Analysis of group demographics and questionnaire data

fMRI analysis groups (*n* = 14)	ADD	LSDD	HSDD	IBS
Female	6 (42.9%)	8 (57.1%)	11 (78.8%)	11 (78.8%)
Age (years), median (IQR)	61.5 (60–66.5)	62 (57.75–66.5)	54.5*^,+^ (51–58.75)	46.5**^,+^ (41.25–47.75)
Previous diverticulitis	0	50%	35.7%	0
Past psychiatric history	28.6%	7.1%	21.4%	42.9%
BMI (kg/m^2^), median (IQR)	26.5 (23.4–28.1)	28.0 (25.3–31.6)	30.5 (24.2–31.4)	24.4^+^ (23.5–27.8)
Gastrointestinal symptoms				
Days/month of pain (<24 h), median (IQR)	0	3* (0–11)	15*** (5–28)	7.5*** (3.3–12)
Pain duration (h), median (IQR)	0	1.5* (0–5)	6** (3.4–2.4)	2.5** (0.8–12)
Sensory testing				
Median VAS temperature HAND (°C) (range)	45.4 (39.5–49)	43.8 (41.5–47.5)	43.8 (41–48)	43.8 (40–49)
Median VAS temperature FOOT (°C) (range)	45.5 (40–48)	43.5 (42–49.5)	43.8 (40.5–47.5)	44.5 (41.5–48.5)
Questionnaire data				
PHQ‐12 SS, median (IQR)	2.5 (2–3)	4* (4–5)	8***^,+++^ (8–9)	8*^,+^ (5–8.75)
HAD: anxiety, median (IQR)	5.5 (3.75–7)	5.5 (3.25–7)	8.5*^,+^ (6.25–11.75)	7 (3–10)
HAD: depression, median (IQR)	2.5 (1–3)	2 (1.25–3.75)	6.5*^,+^ (4–8.75)	4.5 (2–5.75)
Pain catastrophizing score, median (IQR)	11 (2.5–14.5)	3.5 (1.25–15.5)	14.5^+^ (10.5–17.75)	11 (7.75–17.75)

ADD *vs* group *p < 0.05, **p < 0.001, ***p < 0.0001. LSDD *vs* group ^+^ *p* < 0.05, ^++^ *p* < 0.001, ^+++^ *p* < 0.0001. ADD, Asymptomatic diverticular disease; LSDD, Low somatization score diverticular disease; HSDD, High somatization score diverticular disease; IBS, Irritable bowel syndrome; IQR, Interquartile range.

### Questionnaires results

#### Participant gastrointestinal symptoms and psychological questionnaire results

Demographics and gastrointestinal pain symptoms in each group are shown in Table 2. The LSDD, HSDD, and IBS groups PHQ‐12 SS scores were significantly higher (paired *t*‐test) than the ADD group, but the HSDD and IBS were not significantly different. Paired *t*‐test of BMI demonstrated a significant difference between the LSDD and IBS groups (*p* = 0.02), but no differences were found between the other groups. The HSDD group had significant higher PCS and HAD scores compared to the ADD and LSDD groups, but not the IBS group. This suggests a similarity between the IBS and HSDD groups in terms of somatization.

### fMRI results

#### Anticipation and pain effects

**Figure 3:**
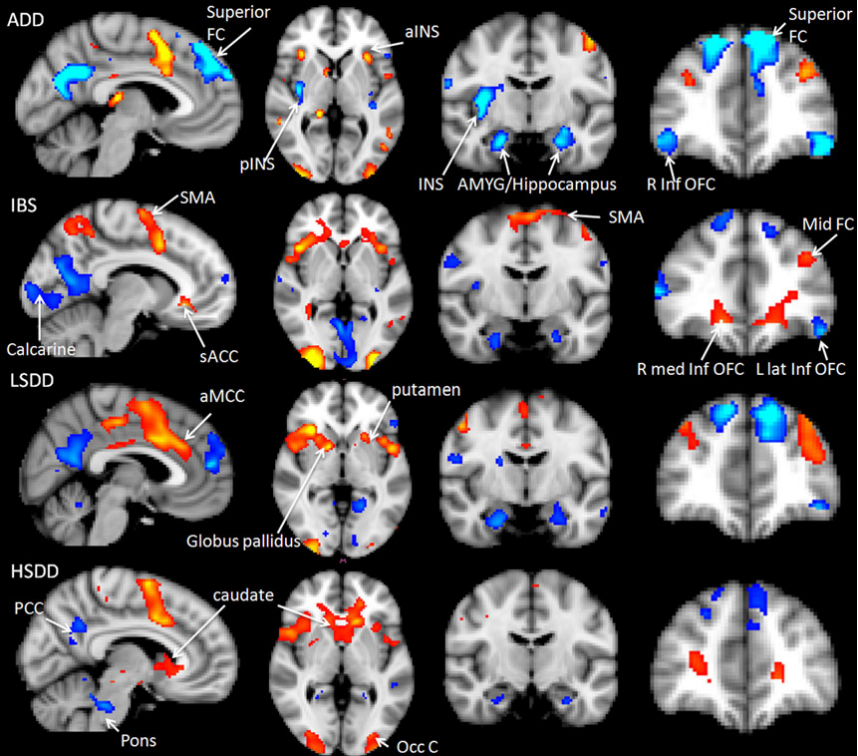
Global BOLD effects for anticipation of a painful stimulus applied to the left hand or left foot for the ADD, LSDD, HSDD and IBS groups. Negative BOLD effects are depicted in the blue color spectrum while positive BOLD effects are show in the red–yellow spectrum.

Second‐level analysis demonstrated robust increases and decreases in cortical responses to the anticipation of subsequent painful heat at the MPT stimulus for each group (Fig. 3). Global anticipation effects in the cue period to both left hand and foot stimulation (uncorrected *p* < 0.001, cluster threshold 5) revealed deactivation of the right posterior insula (pINS) and the PFC in the ADD and LSDD groups, areas which are responsible for somatosensory pain‐processing pathway and DNIC areas, respectively (Fig. 3). For all groups, increased activation was observed in affective pain regions, including the anterior insula (aINS) and left ACC in both SDD and IBS groups.

### Inter‐group differences in cortical response to anticipation

**Figure 4:**
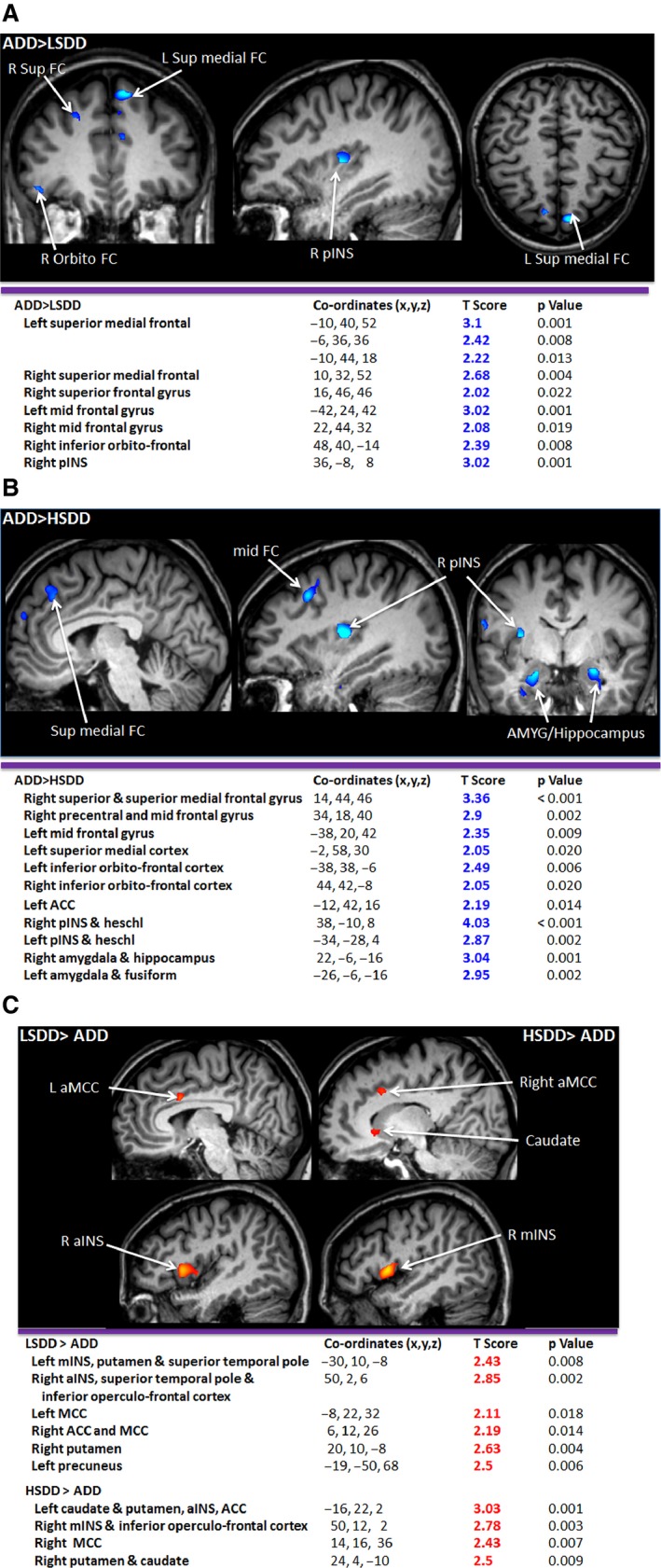
Areas which have statistically more significant deactivation in the ADD group than (A) the LSDD group and (B) the HSDD group. (C) shows areas for which positive activations in the LSDD and HSDD groups is statistically more significant than the ADD group. Deactivations are depicted in the blue color spectrum while activations are show in the red–yellow spectrum.

**Figure 5:**
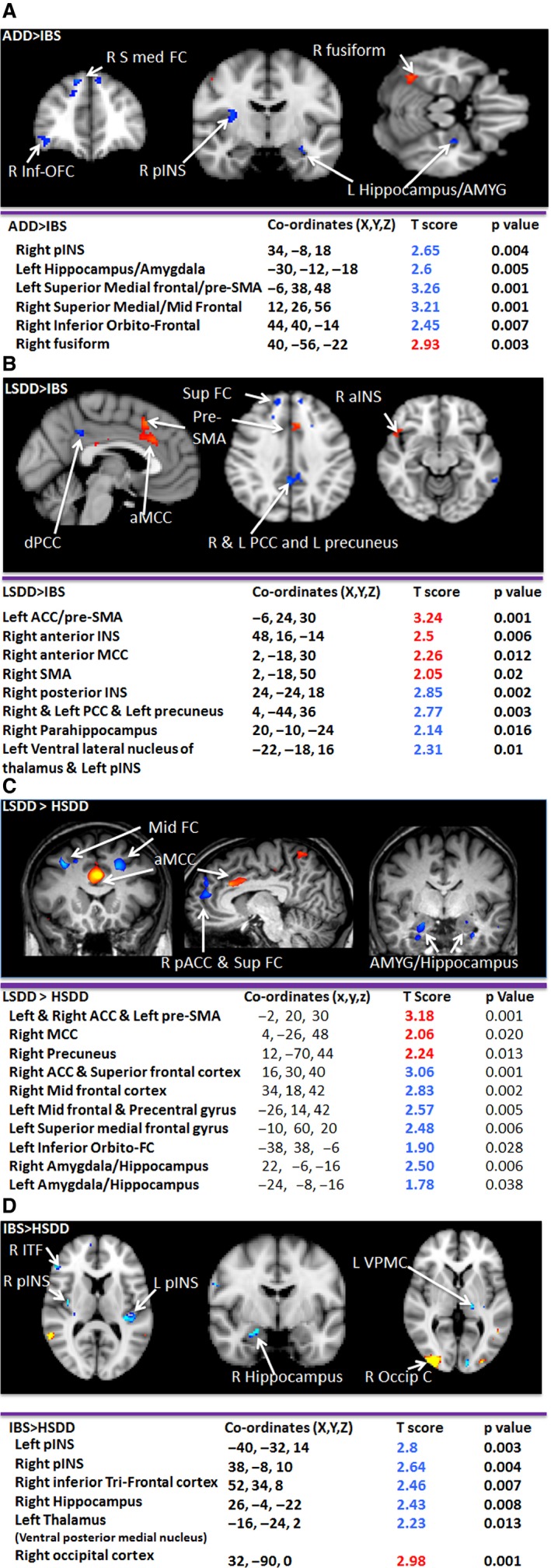
Inter‐Group Analysis: Areas which have statistically more significant deactivation or activation during the cue stimulus in the (A) LSDD than the HSDD group, (B) the LSDD than the IBS group, (C) the ADD than the IBS group, and (D) the IBS compared to the HSDD group. Deactivations are depicted in the blue color spectrum while activations are show in the red–yellow spectrum.

Inter‐group analysis of the cortical regions associated with anticipation was performed to assess differences between the groups in key pain‐processing regions (uncorrected *p* < 0.05 voxel threshold 5) (Figs 4 and 5, Table S1).

#### Somatosensory pain‐processing regions (pINS, THAL)

Greater deactivation was found in the right pINS in the ADD compared to the LSDD (Fig. 4A) and IBS groups (Fig. 5A), and in the bilateral pINS in the ADD compared to the HSDD group (Fig. 4B). Greater right pINS deactivation was also seen in the LSDD compared to the IBS group (Fig. 5B) but the difference between the LSDD and HSDD groups was not significant. Interestingly, the HSDD group showed less bilateral pINS deactivation compared to the IBS group. The HSDD group also demonstrated less deactivation in the left ventral posterolateral nucleus of the thalamus compared to the ADD. In the LSDD group, greater deactivations were found in the left ventral lateral nucleus of the thalamus and pulvinar compared to the IBS group (Fig. 5B). No difference in thalamic activity was identified between the SDD groups.

#### Emotional pain processing regions (aINS, mINS, ACC, MCC, AMYG, parahippocampus)

Greater positive activation was identified in several emotional pain processing areas in the SDD and IBS groups compared to the ADD group. Greater activation in the bilateral aINS/mINS in the LSDD and left aINS in the IBS and HSDD groups was seen compared to the ADD group (Fig. 4C), and greater aINS/mINS activation in the LSDD compared to IBS and HSDD groups. Between all the groups, different areas of the mid‐cingulate showed activation. Notable differences were identified in the left ACC in the LSDD group compared to the HSDD group (Fig. 5C).

Greater deactivation was seen in the AMYG in the ADD compared to the HSDD group (Fig. 4B) and the hippocampus in the ADD compared to the HSDD and IBS groups (Fig. 5A). Interestingly no difference was detected in these regions between the ADD and LSDD groups (Fig. 4A). However, greater left AMYG and right hippocampus deactivation was seen in the LSDD compared to the HSDD group (Fig. 5C). Hippocampal and parahippocampus deactivation was greater in the ADD and LSDD groups than the IBS group (Fig. 5A). Greater hippocampal deactivation was found in the IBS compared to the HSDD group.

#### DNIC regions (PFC)

Greater deactivation was seen in the superior and superior medial frontal and parts of the medial frontal gyrus and orbito‐PFC in the ADD compared to the IBS, LSDD, and HSDD groups (Figs 4 and 5A). Small areas of mid frontal gyrus activation were seen in the ADD compared to the HSDD group and in the LSDD group compared to the IBS and HSDD groups. In comparison greater activation of the superior frontal and inferior orbito or operculo frontal cortex were seen in the IBS and HSDD groups compared to the ADD groups (see Tables S1 and S2).

### Covariates analysis of cue stimulus

Increasing PHQ‐12 SS scores were found to correlate with greater deactivation of the parahippocampus and greater positive activation in the mPFC and MCC and right AMYG/hippocampus (Table S2). In contrast, decreasing PHQ‐12 SS scores correlated with greater deactivation of the AMYG, hippocampus, pINS, and the lateral and orbito‐PFC.

The anxiety component of the HAD score positively correlated with the activation of emotional processing regions, including greater left aINS and right AMYG activity, while decreasing anxiety scores correlated with deactivation of the right AMYG and left hippocampus. In comparison, the depression component of the HAD score positively correlated with greater activation of the left hippocampus, ACC and PCC, while decreasing depression score correlated with greater activation of the MCC and greater deactivation of the ACC, right AMYG, parahippocampus and left hippocampus. Similar findings were identified for the increasing PCS scores which correlated with parahippocampus deactivation, while decreasing PCS scores correlated with left aINS, ACC and PFC activation, and hippocampal deactivation.

## Discussion

This study has demonstrated changes during anticipation in the somatosensory and emotional pain‐processing regions and DNIC regions, which alter across ADD, LSDD, HSDD, and IBS groups. Greater deactivations were seen in the pINS in the ADD compared to the LSDD, HSDD, and IBS groups (Fig 4). The pINS is key in discriminative‐sensory pain processing.^43^ Deactivation of the pINS during anticipation has been identified in healthy volunteers compared to IBS patients,^21^ which supports our findings that greater deactivations are characteristic of the group with lesser symptoms. Greater pINS deactivation is correlated with decreasing PHQ‐12 SS score but it did not correlate with scores of catastrophizing, anxiety, or depression. Similar findings have been reported in other studies of somatization, although in this study, significant correlations with somatization score was not confirmed.^44^ A reduction in pINS, and in aINS, THAL, and hippocampal responses, in IBS patients has been identified after hypnotherapy compared to educational interventions,^45^ which may be related to better coping methods in treated individuals, and may explain the link with somatization.

Greater deactivation of the thalamus was found in the ADD and IBS groups compared to HSDD group, and in the LSDD group compared to IBS group. The thalamus is also a key area in the somatosensory pain‐processing pathway, forming part of the spinothalamic tracts with fibers running to the pINS^46^ and motor responses to pain. In our study the ventral posterolateral thalamic nucleus, which receives signals from the spinothalamic tracts and projects to the primary somatosensory cortex,^47^ was demonstrated to deactivate in the ADD compared to the HSDD group. This observation agrees with healthy volunteer studies of placebo analgesia in rectal pain, where responders to placebo analgesia were noted to have a reduction in thalamic response^48^ and animal models.^49^ In the LSDD group, deactivation of the ventral lateral nucleus and pulvinar were observed compared to the IBS group. The ventral lateral nucleus outputs projections to the motor cortex, and may be involved in motor responses to pain. The pulvinar has connections to the ACC, prefrontal cortex, and AMYG,^50^, ^51^, ^52^ which has been shown have heightened connectivity with the pINS during pain anticipation in patients with major depressive disorder (MDD).^53^ Similarly positive correlation between scores for neuroticism and thalamus activity during pain anticipation in healthy volunteers has also been identified.^54^ Deactivation in the pINS, pulvinar, and posterolateral thalamus is suggestive of preparation for anticipated pain in our ADD and LSDD groups, which is reduced or absent in our chronic pain groups.

The affective processing regions demonstrated greater activation during anticipation in our IBS and SDD groups compared to the ADD group (Fig. 4). The aINS is a key area in affective pain processing and is important for interoception,^55^ emotional awareness,^55^ and risk prediction.^56^ Greater activation of the aINS during anticipation has also been found in healthy volunteers^57^ and other pain groups, such as anorexia nervosa and IBS.^58^, ^59^ Anxiety can influence INS activity during anticipation^60^, ^61^ as was also demonstrated in our study (Table S2). This suggests a greater emotional response to impending pain processing in our SDD and IBS groups.

There was a lack of deactivation in the MCC and ACC in our chronic pain groups (HSDD and IBS) compared to the ADD group (Fig. 4). The ACC and/or MCC have previously been identified in studies of healthy volunteers^25^, ^57^ and IBS pain anticipation^21^, ^62^ and it is thought that cingulate cortex activation may be related to attention,^63^ affective processing of painful stimuli, reward probability and risk,^64^ and information flow between somatic and emotional brain regions.^65^, ^66^ A reduction in ACC and MCC activation during anticipation of pain has also been identified in IBS patients during placebo^67^ and longitudinal studies with repeated stimulations.^62^ Anxiety also influences MCC activation during ‘cued’ sham gastric distensions.^68^ This may be why deactivation in the MCC was negatively correlated with HAD anxiety score in our study. The ACC may also be important before the anticipation phase, as resting‐state functional connectivity between the ACC and medial PFC has been correlated with changes in ‘cued’ pain score.^69^ Thus, greater ACC activation and a failure to deactivate the MCC during anticipation may suggest alteration in connectivity between the somatic and emotional brain regions in our chronic pain groups.

Differences were identified in other affective areas, with greater deactivations in AMYG and hippocampal areas in the ADD group compared to the LSDD, HSDD, and IBS groups (Fig 4). These areas have been reported by other groups in healthy volunteers during pain anticipation.^21^, ^70^ In our study IBS showed less deactivation in the AMYG and hippocampus than LSDD and ADD groups. Several studies in IBS have reported greater AMYG or hippocampal activation during anticipation^59^, ^71^ which decreases with increased study familiarity^62^ (longitudinal studies) and reduced anxiety. Deactivations of the AMYG and hippocampus were related to both PHQ‐12 SS, anxiety, depression, and PCS scores. Higher PHQ‐12 SS scores were associated with less deactivation of the right amygdala and bilateral hippocampus regions, suggesting that these areas play an important role in controlling pain sensation. The PHQ‐12 SS and PCS scores also positively correlated with the deactivation of the bilateral parahippocampus and positive activation of the right AMYG/hippocampus. Anxiety score was positively correlated with greater left amygdala activity, and both depression and anxiety score were negatively correlated with right AMYG and left hippocampal deactivation. These findings agree with anticipation studies in MDD patients, where AMYG activity was correlated with perceived helplessness scores.^72^ Minimal differences in hippocampal activity between high‐ and low‐anxiety states have been identified in people with chronic daily symptoms.^43^ Thus, greater deactivation in the AMYG and hippocampus may represent reduced anxiety and somatization in the ADD and LSDD in the face of expected pain, that is, better coping, compared to the IBS and HSDD groups. This may underlie some of the differences in anticipatory brain responses^71^ and underlying pathophysiology and treatment strategies for our LSSD and HSDD groups, and gives light on the differences in underlying pathophysiology and possible treatment strategies.

Descending noxious inhibitory control regions showed important differences across patient groups. The DNIC contains many regions including the hypothalamus, AMYG, ACC, periaqueductal gray, and DLPFC.^73^ The role of the frontal cortex in the DNIC is complex and not well understood. The DLPFC is a functional area mainly found in the medial frontal gyrus (mPFC), but can include parts of the superior frontal gyrus (Brodmann's areas 8, 9, 10, and 46).^74^ The mPFC/DLPFC is thought to aid control over attentional, emotional, and descending inhibitory or facility processes in pain.^75^ The mPFC and ACC also interact with the AMYG, PAG, and nucleus accumbens.^76^ In our study, greater superior, superior medial, and mPFC (which includes the DLPFC), orbito‐FC, and AMYG deactivation was seen in the ADD and the LSDD group compared to the HSDD and IBS groups. This agrees with a meta‐analysis of pain anticipation, where deactivations of the superior frontal gyrus were identified.^77^ In comparison, greater superior frontal gyrus, inferior orbitoFC, and MCC activation was identified in the HSDD and IBS groups compared to the ADD. This again agrees with other studies where activation in the DLPFC was identified during pain anticipation in patients with fybromyalgia, MDD, and recovering anorexics.^58^, ^78^ Descending noxious inhibitory control regions have been observed to be absent in IBS groups compared to healthy controls,^21^ which may contribute to visceral hypersensitivity in these patients. Regions of the Superior, superior medial, and/or medial frontal gyrus deactivations were also correlated with decreasing PHQ‐12 SS, PCS, and depression scores in our study, which has also been demonstrated in healthy volunteer studies of self‐reported anxiety.^75^ This change in the PFC activity may be important in future studies assessing the effect of medications in SDD, as anticipatory PFC activity can predict greater symptom improvement after 5HT3R antagonist Alosetron in IBS patients.^78^, ^79^

### Limitations

Firstly, the participants for this study were recruited through clinics and advertisements and no attempt was made to age or sex match the groups resulting in groups with characteristic ages and sex for each medical condition. No attempt was made to control for stage of menstrual cycle,^80^, ^81^ oral contraceptive, testosterone levels,^82^, ^83^, ^84^ or hormone replacement therapy use,^85^, ^86^ for the duration of chronic pain symptoms or for periods of pain exacerbation or remission due to practical considerations. Although this increases the generalizability of our results as it more accurately reflects the patient population, we also acknowledge that there are known differences in fMRI pain processing between genders,^87^, ^88^ age^89^, ^90^, ^91^ groups, and hormonal mechanisms,^82^, ^83^, ^84^, ^85^, ^86^ which may have influences our results. However, we did control for factors, such as increased risk of cardiovascular disease, which may alter blood flow dynamics and cause confounding and we also excluded patients on medications which are thought to the effect of blood flow dynamics such as antiepileptic, antipsychotic, and anxiolytic medications, such as gabapentin.^28^, ^92^, ^93^ Even with these measures, the age difference between the groups maybe important as age‐related changes in brain volume in areas involved in pain processing^31^, ^89^ and DNIC responses^31^ have been identified. Similar changes to brain volume and pain processing have also been associated with pain duration, which may also have affected our results.^94^, ^95^, ^96^ Visceral and cutaneous hypersensitivity is known only to affect a subset of patients with IBS.^97^ In our study, we did not investigate or select our patients based on this phenomenon. This again may have influenced our results as we may not have had sufficient power to detect altered cutaneous sensitivity between our groups.

### Clinical implications

This study has demonstrated differences in anticipated pain processing between diverticular patients with low and high somatization score which has implications for clinical management. There were similar cortical patterns of activity between the HSDD and IBS in whom central abnormalities of pain processing predominate. Our results are compatible with our hypothesis that LSDD results from a predominantly peripheral pathophysiological pain process, while HSDD is predominantly central.^22^ Several studies in healthy volunteers and/or patients with central changes in pain processing, such as IBS, have shown responses to centrally acting medications or techniques such as hypnotherapy or meditation techniques.^97^, ^98^ These may be useful treatments which should be evaluated in patients with SDD with high somatization scores. The PHQ‐12 SS scale is a simple 12‐item scale which could be readily administered in the clinic to identify such patients who may have suboptimal outcomes with surgical intervention but might respond to psychological therapies or medical treatments.

## Conclusions

This is the first study to identify differences in anticipatory pain processing between ADD and SDD. Our study suggests that by classifying SDD patients into high‐ and low‐somatization groups, it is possible to identify altered anticipatory responses to thermal pain. This suggests underlying differences in pain pathophysiology in these groups, and that SDD patients need individualized treatment strategies to target the causes of their chronic pain.

## Funding

This study was funded by the Wellcome Trust Research Training Fellowship awarded to J Smith (WT086609MA).

## Disclosure

There are no conflicts of interest or competing interests declared for any of the authors.

## Author Contribution

JS, DH, LM, RS, SF, PG designed the study; JS performed the research; JS, LM, SF analyzed the data; JS, DH, LM, RS, SF, PG wrote and/or reviewed the paper.

## Supporting information


**Table S1** Intergroup analysis.
**Table S2** Covariate analysis.
