# Supplementary material for: Anticipation of thermal pain in diverticular disease
Source: Neurogastroenterol Motil. 2016 Mar 11;28(6):900–13. doi: 10.1111/nmo.12790 (PMC4879512; doi:10.1111/nmo.12790)
Supplement: Supplementary file 1 — Table S1 Intergroup analysis. Table S2 Covariate analysis. [file NMO-28-900-s001.docx]

**Supplemental Tables**

Table A1:Intergroup analysis

1. Tabulated results of 2 sample t test comparing activations and deactivations between the ADD and LSDD, HSDD and IBS groups during anticipation (CUE) phase. Uncorrected p<0.05, voxel threshold 5

| **Brain regions** |  | |  | **Activations** | | | | | **Deactivations** | | | | | | | | |
| --- | --- | --- | --- | --- | --- | --- | --- | --- | --- | --- | --- | --- | --- | --- | --- | --- | --- |
| **ADD>LSDD** |  | |  | **Cluster Size** | | **T-Score** | **X,Y,Z** | **BA** | **Cluster Size** | | | **T-Score** | | **X,Y,Z** | | | **BA** |
| Insula | Post. | | R |  | |  |  |  | 63 | | | **3.02** | | 36,-8,8 | | |  |
| Frontal | Sup Med. | | R |  | |  |  |  | 64 | | | **2.68** | | 10,32,52 | | |  |
|  |  | | L |  | |  |  |  | 58 | | | **3.1** | | -10,40,52 | | |  |
|  | Med | | R |  | |  |  |  | 34 | | | **2.08** | | 22,44,32 | | |  |
|  |  | | L |  | |  |  |  | 45 | | | **3.02** | | -42,24,42 | | | 9 |
|  | Orbito | | R |  | |  |  |  | 15 | | | **2.39** | | 48,40,-14 | | |  |
| **LSDD>ADD** | | | | | | | | | | | | | | | | | |
| Insula | Mid | | L | 172 | | **2.43** | -30,10,6 | 28 | |  | |  | |  | | |  |
|  | Ant. | | R | 537 | | **2.85** | 50,2,-8 | 47 | |  | |  | |  | | |  |
| Cingulate | Mid | | R | 75 | | **2.19** | 6,12,26 | 32,33, | |  | |  | |  | | |  |
|  |  | | L | 94 | | **2.11** | -8,22,32 |  | |  | |  | |  | | |  |
| Frontal | Sup. | | L |  | |  |  |  | | 44 | | **2.01** | | -22,26,38 | | |  |
|  | Mid | | R | 6 | | **1.76** | 38,46,16 | 10 | |  | |  | |  | | |  |
| Putamen |  | | R | 141 | | **2.63** | 20,10,-8 |  | |  | |  | |  | | |  |
| Lat. Post Nuc. |  | | L |  | |  |  |  | | 5 | | **2.07** | | -20,-20,14 | | |  |
| **ADD>HSDD** |  | |  |  | | | | | |  | | | | | | | |
| Insula | Post. | | R |  | |  |  |  | | 266 | | | **4.03** | | 38,-10,8 | | 13 |
|  |  | | L |  | |  |  |  | | 50 | | | **2.87** | | -34,-28,-28 | |  |
| Frontal | Sup. | | R |  | |  |  |  | | 577 | | | **3.36** | | 14,44,46 | |  |
|  | Sup Med. | | L |  | |  |  |  | | 29 | | | **2.05** | | -2,58,30 | |  |
|  | Mid | | L | 8 | | **1.99** | -30,32,28 |  | | 80 | | | **2.35** | | -38,20,42 | |  |
|  | Orbito | | R |  | |  |  |  | | 29 | | | **2.05** | | 44,42,-8 | |  |
|  |  | | L |  | |  |  |  | | 76 | | | **2.49** | | -38,38,-6 | |  |
| Thalamus | Vent.. Post . Lat. Nuc. | | L |  | |  |  |  | | 5 | | | **2.06** | | -18,-22,0 | |  |
| Amygdala |  | | L |  | |  |  |  | | 143 | | | **2.95** | | -26,-6,-16 | |  |
| Hippocampus |  | | R |  | |  |  |  | | 108 | | | **3.04** | | 22,-6,-16 | |  |
| **HSDD>ADD** | | | | | | | | | | | | | | | | | |
| Insula | Ant. | L | | 198 | | **3.03** | -16,22,2 | 24 | |  | | |  | |  | |  |
| Cingulate | Mid | R | | 23 | | **2.43** | 14,16,36 | 32 | |  | | |  | |  | |  |
| Frontal | Sup. | L | | 6 | | **2.27** | -14,-8,70 |  | |  | | |  | |  | |  |
|  | Sup. | R | | 7 | | **1.94** | 28,44,8 |  | |  | | |  | |  | |  |
|  | Operculo & Insula (mid) | R | | 231 | | **2.78** | 50,12,2 | 44 | |  | | |  | |  | |  |
| Putamen |  | R | | 281 | | **2.35** | 24,4,-10 |  | |  | | |  | |  | |  |
| **ADD>IBS** |  |  | |  | | | | | |  | | | | | | | |
| Insula | Post. | R | |  | |  |  |  | | 197 | | **2.65** | | | 34,-8,18 | 13 | |
| Cingulate | Mid | R | | 65 | | **2.23** | -2,-2,28 | 23 | | 59 | | **2.12** | | | 2,-44,34 | 31 | |
| Frontal | Sup Med. | R | |  | |  |  |  | | 453 | | **3.21** | | | 12,26,56 |  | |
|  |  | L | |  | |  |  |  | | 430 | | **3.26** | | | -6,38,48 | 6 | |
|  | Orbito | R | |  | |  |  |  | | 41 | | **2.45** | | | 44,40,-14 | 11 | |
| Hippocampus |  | L | |  | |  |  |  | | 79 | | **2.6** | | | -30,-12,-18 |  | |
| Parahippocampus |  | R | |  | |  |  |  | | 10 | | **1.81** | | | 22,-10,-22 | 34 | |
|  |  | R | |  | |  |  |  | | 11 | | **1.93** | | | 34,-18,-22 |  | |
| **IBS>ADD** | | | | | | | | | | | | | | | | | |
| Insula | Ant. | L | | | 10 | **1.87** | -38,18,6 | 13 | | |  |  | | |  |  | |
| Cingulate | Mid | R | | | 19 | **1.87** | 12,20,32 |  | | |  |  | | |  |  | |
| Frontal | Sup. | R | | | 31 | **2.42** | 24,48,16 |  | | | 19 | **1.97** | | | 14,62,22 |  | |
|  | Orbito | R | | | 119 | **2.07** | 20,28,-10 |  | | |  |  | | |  |  | |
|  | Inf. Operculo | R | | | 151 | **3.3** | 46,16,0 |  | | |  |  | | |  |  | |
|  | SMA | L | | | 87 | **2.49** | 0,-10,68 | 6 | | |  |  | | |  |  | |
|  |  | L | | | 19 | **2.36** | -12,6,48 | 24 | | |  |  | | |  |  | |
|  |  | R | | | 43 | **1.98** | 14,6,58 | 6 | | |  |  | | |  |  | |
| Amygdala |  | R | | |  |  |  |  | | | 939 | **2.85** | | | 12,-60,8 |  | |

(ii) Tabulated results of 2 sample t test comparing activations and deactivations between the IBS and LSDD, HSDD groups during anticipation (CUE) phase.

| **IBS>HSDD** |  |  | **Activations** | | | | **Deactivations** | | | |
| --- | --- | --- | --- | --- | --- | --- | --- | --- | --- | --- |
| **Brain regions** |  |  | **Cluster Size** | **T-Score** | **X,Y,Z** | **BA** | **Cluster Size** | **T-Score** | **X,Y,Z** | **BA** |
| Insula | Post. | R |  |  |  |  | **15** | **2.64** | **38,-8,10** |  |
|  |  | L |  |  |  |  | **173** | **2.8** | **-40,-32,14** | **41** |
| Frontal | Sup Med. | R |  |  |  |  | **47** | **2.23** | **12,60,14** |  |
| Thalamus | Lat. Post Med. Nuc. | L |  |  |  |  | **13** | **2.23** | **-16,-24,2** |  |
| Hippocampus |  | R |  |  |  |  | **30** | **2.43** | **26,-4,-22** |  |
| **HSDD>IBS** | | | | | | | | | | |
| Cingulate | Mid | R |  |  |  |  | **77** | **2** | **2,-44,36** | **7,31** |
| Frontal | Sup. | R |  |  |  |  | **44** | **2.53** | **22,50,34** |  |
|  |  | L |  |  |  |  | **36** | **2.38** | **-10,28,54** |  |
| Caudate |  | L | **77** | **2.23** | **-10,14,0** |  |  |  |  |  |
| Putamen |  | R | **10** | **1.91** | **24,6,-2** |  |  |  |  |  |
| **IBS>LSDD** |  |  |  | | | |  | | | |
| Cingulate | Ant | R | **76** | **2.36** | **30,26,-14** |  |  |  |  |  |
| Caudate |  | R | **38** | **2.15** | **2,18,6** |  |  |  |  |  |
| Caudate |  | L | **23** | **1.86** | **-20,32,-2** |  |  |  |  |  |
| **LSDD>IBS** | | | | | | | | | | |
| Insula | Post. | R |  |  |  |  | **55** | **2.85** | **24,-24,18** | **13** |
|  | Mid | R | **100** | **2.5** | **48,16,-14** | **38** |  |  |  |  |
|  | Ant. | R | **24** | **2.01** | **32,20,-2** |  |  |  |  |  |
| Cingulate | Mid | R | **83** | **2.26** | **2,-18,30** | **23** | **279** | **2.77** | **4,-44,36** | **31** |
|  | Ant | L | **449** | **3.24** | **-6,24,30** | **32** |  |  |  |  |
| Frontal | Sup. | R |  |  |  |  | **291** | **2.68** | **14,48,34** |  |
|  |  | L |  |  |  |  | **155** | **3.14** | **-12,48,30** | **9** |
|  | Sup Med. | L |  |  |  |  | **295** | **2.45** | **-8,28,54** |  |
| Thalamus | Vent. Lat. Nuc | L |  |  |  |  | **24** | **2.31** | **-22,-18,16** |  |
| Hippocampus |  | L |  |  |  |  | **18** | **1.88** | **-32,-14,-22** |  |
| Parahippocampus |  | R |  |  |  |  | **75** | **2.14** | **20,-10,-24** | **28** |

1. Tabulated results of 2 sample t test comparing activations and deactivations between the LSDD and HSDD groups during anticipation (CUE) phase.

| **LSDD>HSDD** |  |  | **Activations** | | | | **Deactivations** | | | |
| --- | --- | --- | --- | --- | --- | --- | --- | --- | --- | --- |
| **Brain regions** |  |  | **Cluster Size** | **T-Score** | **X,Y,Z** | **BA** | **Cluster Size** | **T-Score** | **X,Y,Z** | **BA** |
| Insula | Mid | R | 46 | **2.06** | 4,-26,48 | 31 |  |  |  |  |
|  | Ant. | L | 610 | **3.18** | -2,20,30 | 32 |  |  |  |  |
| Cingulate | Ant | R |  |  |  |  | 510 | **3.06** | 16,30,40 |  |
| Frontal | Sup Med. | L |  |  |  |  | 193 | **2.48** | -10,60,20 |  |
|  |  | L |  |  |  |  |  |  |  |  |
|  | Mid | R | 8 | **1.87** | 38,52,14 |  | 151 | **2.83** | 34,18,42 |  |
|  |  | L | 39 | **2.28** | -30,42,36 |  | 245 | **2.57** | -26,14,42 | 9 |
|  |  | L | 62 | **2.15** | -36,48,20 |  |  |  |  |  |
|  | Orbito | R |  |  |  |  | 14 | **1.9** | -38,38,-6 |  |
| Lat. Globus Pallidus |  | R | 6 | **1.99** | 14,2,6 |  |  |  |  |  |
|  |  | R |  |  |  |  | 7 | **1.81** | 18,-24,2 |  |
| Amygdala |  | L |  |  |  |  | 5 | **1.78** | -24,-8,-16 |  |
| Hippocampus |  | R |  |  |  |  | 87 | **2.5** | 22,-6,-16 |  |
|  |  | R |  |  |  |  | 6 | **1.84** | 34,-10,-26 |  |
| **HSDD>LSDD** | | | | | | | | | | |
| Cingulate | Mid | L | 5 | **1.85** | -12,2,40 |  |  |  |  |  |
|  | Ant | L | 42 | **2.02** | -8,30,0 |  |  |  |  |  |
| Caudate |  | R | 18 | **1.86** | 4,16,4 |  |  |  |  |  |
| Caudate |  | L | 5 | **1.87** | -16,22,2 |  |  |  |  |  |

.

**Table A2**: Covariate analysis. Tabulated results of 1 sample t test comparing activations and deactivations of the ADD, LSDD, HSDD and IBS groups during anticipation (CUE) phase and participant PHQ12 scores. Positive correlations: Uncorrected p<0.05, voxel threshold 5. Negative Correlations: Uncorrected p<0.01, voxel threshold 5.

| **A: PHQ12 positive correlations** | | |  | **Activations** | | | | | | | | | **Deactivations** | | | | | | | | | |
| --- | --- | --- | --- | --- | --- | --- | --- | --- | --- | --- | --- | --- | --- | --- | --- | --- | --- | --- | --- | --- | --- | --- |
| **Brain regions** | |  |  | **Cluster Size** | **T-Score** | | | **X,Y,Z** | | | **BA** | | **Cluster Size** | **T-Score** | | | **X,Y,Z** | | | **BA** | | |
| Cingulate | | Mid | **L** | **40** | **2.41** | | | **-14,8,44** | | |  | |  |  | | |  | | |  | | |
|  | |  | **R** | **46** | **2.42** | | | **14,20,36** | | | **24** | |  |  | | |  | | |  | | |
| Amygdala/hippocampus | |  | **R** | **2028** | **4.23** | | | **26,6,-14** | | |  | |  |  | | |  | | |  | | |
| Parahippocampus | |  | **R** |  |  | | |  | | |  | | **406** | **3.53** | | | **18,-42,-4** | | |  | | |
| **Negative Correlations** | | | | | | | | | | | | | | | | | | | | | | |
| Insula | | Post. | **L** |  |  | | |  | | |  | | **46** | **2.69** | | | **-12,-46,26** | | |  | | |
| Cingulate | | Mid | **R** | **52** | **3.14** | | | **4,-26,48** | | | **31** | |  |  | | |  | | |  | | |
| Frontal | | Orbito | **L** |  |  | | |  | | |  | | **28** | **2.85** | | | **-46,34,-14** | | | **47** | | |
| Amygdala | |  | **R** |  |  | | |  | | |  | | **331** | **4.08** | | | **22,-4,-14** | | |  | | |
| Hippocampus | |  | **L** |  |  | | |  | | |  | | **144** | **3.05** | | | **-28,-8,-16** | | |  | | |
| **B: HAD: Anxiety positive correlation** | | |  |  | | | | | | | | |  | | | | | | | | | |
| Insula | | Ant. | **L** | **86** | | | **2.45** | | | **-4,40,0** | | **32** |  | | |  | | |  | | |  |
| Amygdala | |  | **R** | **975** | | | **3.79** | | | **26,4,-14** | | **34** |  | | |  | | |  | | |  |
| **Negative Correlations** | | | | | | | | | | | | | | | | | | | | | | |
| Amygdala | |  | **R** |  | |  | | |  | | |  | **248** | | | **3.37** | | | **24,-4,-16** | | |  |
| Hippocampus | |  | **L** |  | |  | | |  | | |  | **9** | | | **1.73** | | | **-28,-10,-14** | | |  |
| **C: HAD: Depression Positive correlation** | | |  |  | | | | | | | | |  | | | | | | | | | |
| Cingulate | | Post | **L** | **125** | | **2.62** | | | **-6,-36,24** | | | **23** |  | | |  | | |  | | |  |
|  | | Ant | **L** | **513** | | **2.76** | | | **-12,40,0** | | | **32** |  | | |  | | |  | | |  |
| Hippocampus | |  | **L** | **1857** | | **3.8** | | | **-26,20,-18** | | |  |  | | |  | | |  | | |  |
| **Negative Correlation** | | | | | | | | | | | | | | | | | | | | | | |
| Insula | | Mid | **L** | **494** | | | **3.07** | | | **-58,4,-6** | | **22,38** |  | | |  | | |  | | |  |
| Cingulate | | Post | **L** |  | | |  | | |  | |  | **1117** | | | **3.75** | | | **-4,-38,16** | | |  |
|  | | Mid | **R** | **1224** | | | **3.09** | | | **2,10,42** | |  |  | | |  | | |  | | |  |
|  | | Ant | **R** |  | | |  | | |  | |  | **3487** | | | **3.4** | | | **-30,18,36** | | |  |
| Amygdala | |  | **R** |  | | |  | | |  | |  | **971** | | | **3.13** | | | **32,-6,-16** | | | 6 |
| Hippocampus | |  | **L** |  | | |  | | |  | |  | **273** | | | **3.01** | | | **-28,-8,-16** | | |  |
| Parahippocampus | |  | **R** |  | | |  | | |  | |  | **50** | | | **2.29** | | | **34,-34,-16** | | | 36 |
| **D: PCS Positive correlation** | | |  |  | | | | | | | | |  | | | | | | | | | |
| Parahippocampus |  | | **R** |  | | |  | | |  | |  | **24** | | **2.06** | | | **22,-40,-8** | | |  | |
| **Negative Correlation** | | | | | | | | | | | | | | | | | | | | | | |
| Insula | Ant. | | **L** | **126** | | | **2.24** | | | **-28,18,6** | |  |  | |  | | |  | | |  | |
| Frontal | Sup Med. | | **L** |  | | |  | | |  | |  | **30** | | **2.28** | | | **-8,56,2** | | | 10 | |
|  | Mid | | **L** | **137** | | | **2.66** | | | **-32,44,20** | |  | **43** | | **2.01** | | | **-26,20,44** | | |  | |
|  | Orbito | | **L** |  | | |  | | |  | |  | **27** | | **2.37** | | | **-48,32,-14** | | |  | |
| Hippocampus |  | | **R** |  | | |  | | |  | |  | **43** | | **2.41** | | | **26,-16,-18** | | |  | |
